# Complete genome sequence of *Rahnella* sp. PAMC25559, a psychrophilic plant growth-promoting bacterium isolated from an Alpine glacier

**DOI:** 10.1128/mra.01160-25

**Published:** 2025-12-19

**Authors:** Junseok Bang, Byeollee Kim, Yung Mi Lee, Hackwon Do, Jun Hyuck Lee, Tae-Jin Oh

**Affiliations:** 1 Department of Life Science and Biochemical Engineering, Graduate School, SunMoon University 35022 , Asan, South Korea; 2 Genome-based BioIT Convergence Institute, Asan, South Korea; 3 Division of Life Sciences, Korea Polar Research Institute 123591, Incheon, South Korea; 4 Department of Pharmaceutical Engineering and Biotechnology, SunMoon University 35022 , Asan, South Korea; Montana State University, Bozeman, Montana, USA

**Keywords:** *Rahnella *sp., complete genome, plant growth promoting, cold adaptation

## Abstract

We report the complete genome sequence of *Rahnella* sp. PAMC25559, isolated from Zugspitz glacier, Austria. The complete genome assembly was generated, consisting of one chromosome and two putative plasmid sequences. The genome includes biosynthetic gene clusters potentially related to cold adaptation and plant growth-promoting activity.

## ANNOUNCEMENT

While *Rahnella* species from various environments have been studied for their plant growth-promoting (PGP) activity, the genetic basis of PGP activity under cold conditions remains poorly understood (1, 2). *Rahnella* sp. PAMC25559 (PAMC25559), isolated from the Zugspitz glacier (Austria) by the Korean Polar Research Institute and deposited in the Polar and Alpine Microbial Collection (PAMC), was retrieved for genome sequencing. Cells were cultivated in 100 mL of R2A broth at 15°C and 150 rpm until OD_600_ of 1.0, measured with Multiskan SkyHigh Spectrophotometer (Thermo Scientific, USA). DNA was extracted using the DNeasy Blood & Tissue Kit (Qiagen, Germany). High-quality DNA was quantified using the Qubit dsDNA HS Assay Kit (Thermo Fisher Scientific, USA). DNA purity and integrity were assessed by NanoDrop 2000 (Thermo Fisher Scientific, USA) and Tapestation 2200 (Agilent Technologies, USA), showing main peak > 20 kb.

For short-read sequencing, DNA was sheared to ~350 bp fragments using a Covaris S2 system and prepared with the TruSeq DNA Nano kit (Illumina, USA), which includes qPCR and size selection. Library quality was verified using TapeStation 2,200. Sequencing was performed on Illumina NovaSeq (2 × 150 bp), generating 9,847,142 reads with a total of 1,486,918,442 bases. Low-quality reads were filtered using Fastp v20.1 (3). For Oxford Nanopore Technology (ONT) long-read sequencing, high-quality DNA without size selection was used based on QC results to prepare libraries with the 1D ligation (SQK-LSK110) and Native Barcoding Expansion (EXP-NBD104) kits. Sequencing was performed on a GridION with R9.4 flow cells (FLO-MIN106). Reads were basecalled using high-accuracy basecalling mode. The 80,601 raw reads (723.7 Mbp length) were generated and filtered using Filtlong v0.2.1 (https://github.com/rrwick/Filtlong) with the options --min_length 1000 and --keep_percent 95, yielding 52,308 reads with a total length of 687.5 Mbp (*N*_50_ = 18,383 bp; 126.6× coverage). Filtered reads were assembled with the Trycycler pipeline v0.5.5 (4). Contigs assembled from subsampled reads using Flye v2.9.3 (5) were clustered and reconciled into a consensus genome sequence. Polishing was performed with Medaka v2.0.1 (https://github.com/nanoporetech/medaka) for ONT reads and Polypolish v0.6.0 (6) and POLCA of the MaSurca_4.1.0 (7) for Illumina reads. Consequently, a complete genome was assembled with a total length of 5,487,592 bp and an overall GC content of 53.2%. CheckM2 v1.1.0 (8) assessment revealed 100% completeness and 0.12% contamination, indicating high-quality genome assembly. Unless otherwise stated, all software used default parameters and kits followed the manufacturer’s instructions without modification.

**Fig 1:**
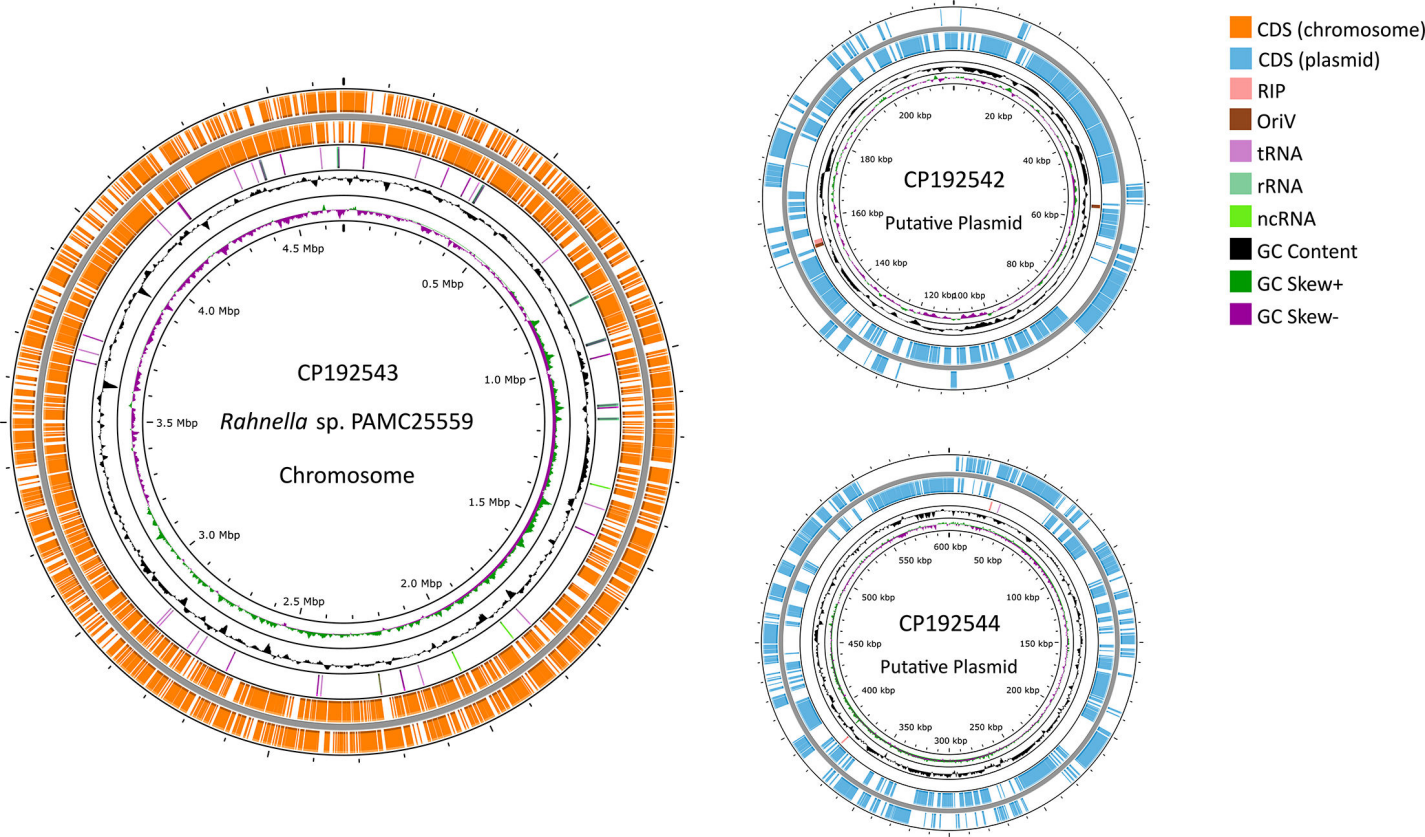
Circular genome map of *Rahnella* sp. PAMC25559: chromosome (left) and two putative plasmids (right). From outside inside: forward and reverse strands showing coding DNA sequences (CDS, orange and sky blue); replication initiation protein (RIP, light pink); replication origin (OriV, brown); tRNA (light purple); rRNA (mint green); ncRNA (lime green); GC content (black); and GC skew (dark green: positive and purple: negative).

Putative replication initiation protein and origin of replication sequences were identified in the two shorter contigs using OriV-Finder (9), suggesting these contigs function as plasmids (10). Annotation was performed using the NCBI PGAP v6.9 (11). Genome features are shown in Table 1, and a circular genome map was generated using Proksee (12) (Fig. 1). With average nucleotide identity (ANI) of 98.26%, PAMC25559 was identified to be most similar to uncultured *Rahnella* (GCA_963515145.1) and *Rahnella* sp. R3 (GCF_042920125.1) based on FastANI v1.34 (13). AntiSMASH v8.0 (14) identified biosynthetic gene clusters: Aryl polyene and desferrioxamine E (NI-siderophore) are potentially associated with cold adaptation and PGP, respectively (15, 16).

**TABLE 1:** Genome features of *Rahnella* sp. PAMC25559

Genome feature	Total	Chromosome (CP192543)	p1PAMC25559 (CP192544)	p2PAMC25559 (CP192542)
Genome size (bp)	5,487,592	4,667,958	600,161	219,437
G+C content (%)	53.20	53.36	53.90	48.00
Contig N_50_ (bp)	4,667,958	4,667,958	600,161	219,437
Contig L_50_	1	1	1	1
Total number of genes	5,008	4,228	511	215
Number of CDSs (total)	4,900	4,181	510	215
Number of CDSs (with protein)	4,882	4,147	499	182
Number of pseudogenes	78	34	11	33
Number of RNA genes	108	107	1	–^*a*^
rRNAs (5S, 16S, 23S)	8, 7, 7	8, 7, 7	–	–
tRNAs	78	78	1	–
ncRNAs	8	8	–	–

^*a*^ – indicates that the feature was not detected/predicted for that replicon (i.e., value = 0).

## Data Availability

The genome project for *Rahnella* sp. PAMC25559 has been deposited in NCBI under BioProject accession number PRJNA1263121 and BioSample accession number SAMN48511426. The raw sequencing reads are available in the Sequence Read Archive (SRA) under the following accession numbers: Nanopore reads, SRX28844765; Illumina reads, SRX28844764. The genome assemblies are available at GenBank under the RefSeq assembly accession number GCF_050632365.1 and the submitted GenBank assembly accession number GCA_050632365.1. The complete nucleotide sequences are available under the following accession numbers: CP192542.1, CP192543.1, and CP192544.1.
